# Discerning primary tumors from metastases in synchronous nasopharyngeal squamous cell carcinoma and cutaneous squamous cell carcinoma: A case report and review of the literature

**DOI:** 10.3892/ol.2014.1915

**Published:** 2014-02-26

**Authors:** RUI AO, RONG FU, DANDAN DONG, XUEQIANG ZHU, HAO LIU, KE XIE

**Affiliations:** 1 Department of Oncology, Sichuan Academy of Medical Sciences, Sichuan Provincial People’s Hospital, Chengdu, Sichuan 610072, P.R. China; 2 Department of Plastic Surgery, Sichuan Academy of Medical Sciences, Sichuan Provincial People’s Hospital, Chengdu, Sichuan 610072, P.R. China; 3 Department of Pathology, Sichuan Academy of Medical Sciences, Sichuan Provincial People’s Hospital, Chengdu, Sichuan 610072, P.R. China

**Keywords:** squamous cell carcinoma, nasopharynx, metastasis

## Abstract

Nasopharyngeal carcinoma is one of the most common types of malignant tumor in Southern China and Southeast Asia, and its etiology is closely associated with Epstein-Barr virus (EBV) infection. Non-keratinizing carcinoma accounts for >95% of all nasopharyngeal carcinoma cases. In addition, metastatic nasopharyngeal carcinoma from other locations in the body is extremely rare. This study reports the case of a 53-year-old female who presented with a lesion on the left nasal alar skin that had slowly developed over a five-year period. A biopsy was obtained and the lesion was histologically diagnosed as cutaneous squamous cell carcinoma (SCC). A nasopharyngeal neoplasm was also detected by 18-fluorine-2-fluoro-2-deoxy-*d*-glucose positron emission tomography/computed tomography and nasopharyngoscopy. A biopsy of the nasopharyngeal neoplasm confirmed a diagnosis of SCC. However, a small EBV-encoded nuclear RNA (EBER) test demonstrated that the nasopharyngeal tumor cells were all negative for EBV. As the majority of nasopharyngeal carcinomas were positive for EBER, it was concluded that the nasopharyngeal carcinoma had metastasized from the cutaneous SCC. A brief review of literature is also presented, in addition to a discussion of the pathogen, epidemiology and diagnosis of cutaneous and nasopharyngeal carcinomas.

## Introduction

Non-melanoma cutaneous cancer is the most common type of malignancy occurring worldwide and consists primarily of basal cell carcinoma and squamous cell carcinoma (SCC) (1). Its occurrence is associated with light exposure, the presence of scars, ethnicity and other factors. Nasopharyngeal carcinoma is one of the most frequent types of malignancy in Southern China and is closely associated with Epstein-Barr virus (EBV) infection (2). The current report presents a case of left nasal alar cutaneous SCC and nasopharyngeal SCC diagnosed concurrently. Based on analysis of histology, epidemiology and etiology of the tumors at the two sites, it was concluded that cutaneous SCC was the primary carcinoma and that it had metastasized to the nasopharynx. A brief literature review is also included on the pathogenesis, epidemiology and diagnosis of cutaneous SCC and nasopharyngeal carcinoma. The patient provided written informed consent for the publication of this study.

## Case report

**Figure 1:**
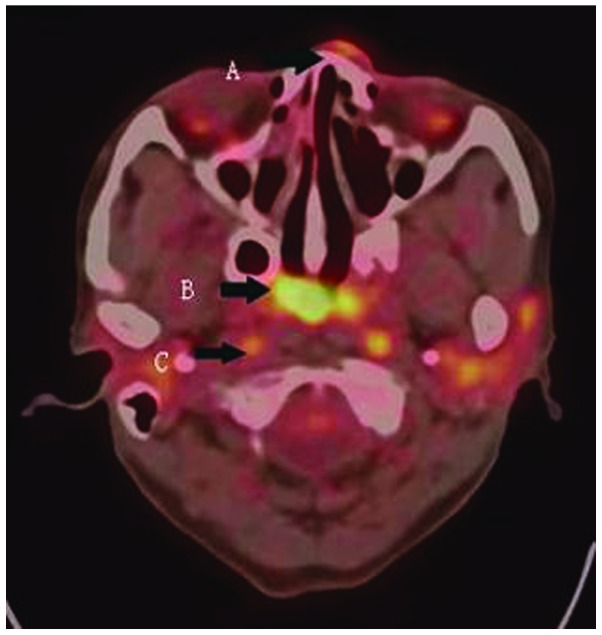
^18^F-FDG uptake in the lesions detected by positron emission tomography/computed tomography. The (A) left nasal alar lesion, (B) nasopharyngeal neoplasm and (C) parapharyngeal lymph nodes demonstrate ^18^F-FDG uptake. ^18^F-FDG, 8-fluorine-2-fluoro-2-deoxy-*d*-glucose.

**Figure 2:**
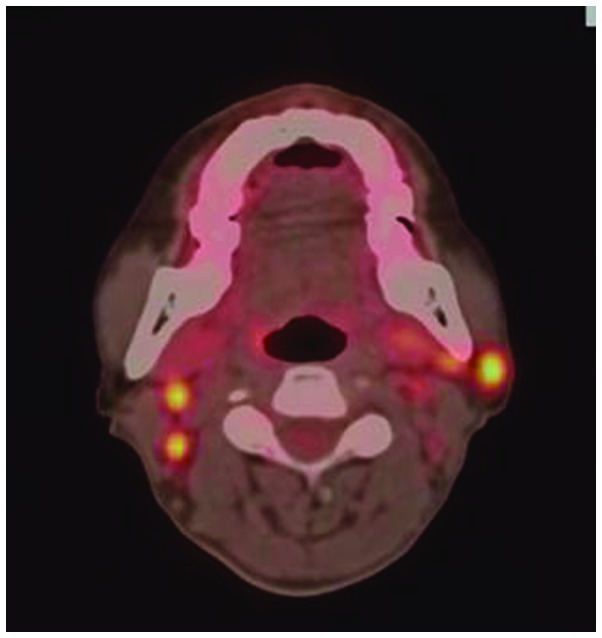
^18^F-fluorodeoxyglucose uptake in cervical lymph nodes as revealed by positron emission tomography/computed tomography.

**Figure 3:**
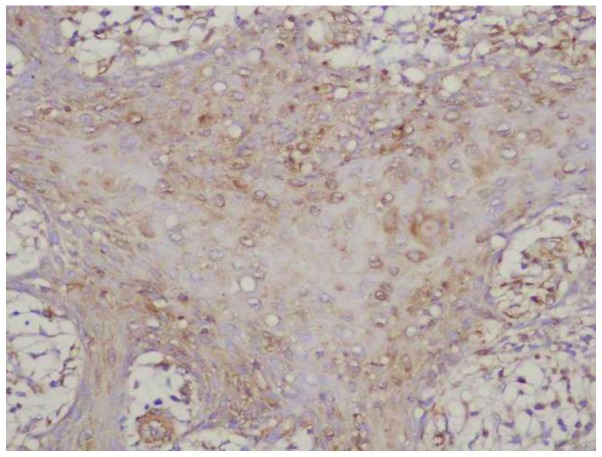
Effect of Epstein-Barr virus-encoded RNA on nasopharyngeal squamous cell carcinoma (magnification, ×200). All cells are negative for EBV.

A 53-year-old female presented with a scar that was accompanied by erosion of the left nasal alar skin. The lesion was 2.5 cm in diameter and had originally developed as a papule, which was 0.3 cm in diameter, five years previously. The patient scratched the papule due to pruritus, which resulted in breakage, and repeatedly scratched the site once the breakage had healed, causing a scar to eventually form. The scar slowly grew during the repeated process of breakage and healing until the patient was admitted to Sichuan Provincial People’s Hospital (Chengdu, China) in November of 2011. The patient consented to whole-body 18-fluorine-2-fluoro-2-deoxy-*d*-glucose (^18^F-FDG) positron emission tomography (PET)/computed tomography (CT) examination, and the results revealed ^18^F-FDG uptake in the left nasal alar skin and the right wall of the nasopharynx. In addition, a number of cervical and parapharyngeal lymph nodes demonstrated ^18^F-FDG uptake (Figs. 1 and 2). The left nasal alar lesion was removed surgically with clear margins, and histological results confirmed that the lesion was cutaneous SCC with keratosis. Examination with a nasopharyngoscope was performed, which revealed a neoplasm on the right wall of the nasopharynx. A biopsy of the neoplasm was conducted, and the pathology results confirmed that the neoplasm was SCC with keratosis. EBV-encoded RNA (EBER) was performed *in situ* in the nasopharyngeal SCC lesion. The nasopharyngeal tumor cells were all negative for EBV (Fig. 3). Based on analysis of histology, etiology and epidemiology of the cutaneous and nasopharyngeal carcinomas, it was concluded that cutaneous SCC was the primary tumor and that it had metastasized to the nasopharynx. The patient refused radiotherapy and chemotherapy, and opted for traditional Chinese medicinal therapy. The patient succumbed to the disease one year after the initial examination.

## Discussion

SCC is an epithelium-derived carcinoma that possesses intercellular bridges or characteristics of keratosis (3). There is a risk of hematogenous and lymphatic metastasis in SCC cases. The patient in the current case report presented with SCC at two sites, namely the left nasal ala and the nasopharynx. This phenomenon is extremely rare, and three possibilities exist that could explain this case: i) The two carcinoma sites were both primary tumors; ii) the tumor of the left nasal ala was a primary tumor that had metastasized to the nasopharynx; or iii) the tumor of the nasopharynx was a primary tumor that had metastasized to the left nasal ala. Based on the findings of this report as discussed below, it was concluded that the primary tumor occurred on the left nose ala and metastasized to the nasopharynx.

Non-melanoma skin cancer consists primarily of basal cell carcinoma and SCC. Of non-melanoma skin cancers, ~80% are basal cell carcinomas, while 20% are SCC (4). The risk factors for the development of cutaneous SCC include exposure to ultraviolet or ionizing radiation, infection with human papillomavirus, ulcers or chronic injury. Chronic scar formation is regarded as one of the most important etiological factors in this disease, and SCC is more likely to develop in skin affected by long-standing ulcers, radiation dermatitis or vaccination scars. Tumors arising at these sites may not be identified for years and, if neglected, carry a substantial risk of metastasis (5). The cutaneous SCC in this patient was detected in the chronic cicatricial areas of the skin and gradually grew over five years. Therefore, it was concluded that the cutaneous SCC on the left nasal alar skin was the primary tumor.

Multiple primary cancers are also relatively uncommon. The incidence of multiple primary cancers has been estimated to be 0.73–11.7% of all cancer patients (6). In a large-scale epidemiological investigation conducted in Japan, only 4% of male and 1% of female cancer patients were expected to develop multiple primary cancers in their lifetime (7). The incidence of synchronous multiple primary cancers is much lower, and only one case of synchronous undifferentiated nasopharyngeal carcinoma and infiltrating ductal carcinoma of the breast has been reported (8). Therefore, the probability that the nasopharyngeal carcinoma in this patient was a synchronous second primary cancer was extremely low.

In addition, nasopharyngeal SCC is relatively uncommon. The incidence of nasopharyngeal carcinoma is 0.5–2/100,000 individuals in Europe and the USA. However, in Southern China, nasopharyngeal carcinoma is endemic, with an incidence of ~25/100,000 individuals (9). The World Health Organization classifies nasopharyngeal carcinoma into three types: Type I, moderately differentiated SCC; type II, non-keratinizing squamous cell, differentiated; and type III, non-keratinizing squamous cell, undifferentiated (10). Of nasopharyngeal carcinoma cases, >95% are classified as non-keratinizing carcinomas (type II) in the nasopharyngeal carcinoma endemic area, and keratinizing SCCs only account for 3–5% of all cases (11). According to a survey conducted by the Sichuan Provincial People’s Hospital, the proportion of SCCs was only 2.4% of all the nasopharyngeal carcinoma cases admitted to the hospital between March 2003 and September 2009 (12).

A marked association between EBV and nasopharyngeal non-keratinizing carcinomas has been reported, although the association between keratinizing SCC and EBV is controversial. However, the majority of researchers in this field still regard keratinizing SCC as being associated with EBV infection. EBERs are small non-coding viral RNAs that are abundantly expressed in cells infected by EBV. Performing EBER detection *in situ* is regarded as one of the most sensitive detection methods for EBV. Zhang *et al* detected the expression of EBER-1 in all keratinizing nasopharyngeal SCC cases assessed by the authors (13). EBER expression has also been detected in nasopharyngeal SCC cases from several geographical regions. For example, in a study by Nicholls *et al*, EBV was detectable in approximately half of patients from Chengdu, which is located in central China (14).

**Figure 4:**
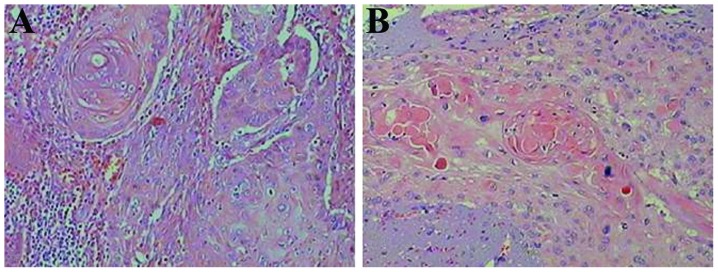
Histopathology of (A) nasopharyngeal SCC and (B) cutaneous SCC with hematoxylin and eosin staining (magnification, ×100). SCC, squamous cell carcinoma.

The patient in the present case report lived in a nasopharyngeal carcinoma-endemic area, but the possibility that the nasopharyngeal carcinoma was a second primary cancer was low due to the uncommon pathological type and the negative EBER test results. More importantly, however, the pathological characteristics of the nasopharyngeal carcinoma in this case were extremely similar to those of the cutaneous SCC. Histological analysis revealed that the two lesions were highly differentiated SCC with keratin pearls (Fig. 4). Based on these findings, it was concluded that the cutaneous SCC was a primary tumor that had metastasized to the nasopharynx.

The risk factors of metastasis of cutaneous SCC include location, size, depth and histological differentiation of the primary tumor, histological evidence of perineural invasion and host immunosuppression. The five-year rate of recurrence of primary cutaneous SCC is 8%, and the five-year rate of metastasis is 5%. In addition, SCCs arising in injured or chronically diseased skin are associated with a risk of metastasis that approaches 40% (5,15). Metastases include regional lymph node metastasis and soft tissue metastasis (STM), where STM is defined as free soft tissue tumor deposits lacking continuity with the primary tumor and without discernible associated lymph node tissue (16). STM can occur by the spread of tumor cells through lymphatic channels that drain the primary tumor or through perineural or vascular routes. We hypothesize that the cutaneous tumor cells of the current patient metastasized to the nasopharynx through lymphatic channels for the following reasons: i) tumors with direct vascular invasion may be more prone to distant spread; ii) there was no clear evidence that the tumor had invaded nerve fibers (nasal alar skin is controlled by the infraorbital nerve and does not pass by the nasopharynx); and iii) ^18^F-FDG PET/CT revealed metastasis to the parapharyngeal lymph nodes near the nasopharynx. It has been demonstrated in an animal model that tumor cells may escape the lymphatic system or travel through small vessels to become free tumor deposits in soft tissues (17). Therefore, we speculate that the tumor cells of this patient may have escaped from lymphatic channels and been deposited in the nasopharynx to form a metastatic tumor.

Metastasis of nasopharyngeal carcinomas is extremely rare, which may partly be due to the fact that the nasopharynx is not a suitable environment for the growth of metastatic tumors. It is also possible that the nasopharynx is well concealed and prevents sufficient detection of metastatic lesions. To the best of our knowledge, this is the first case report describing a case of cutaneous SCC metastasizing to the nasopharynx [only lung cancer metastasis to the nasopharynx has been previously reported (18)]. Therefore, this report may improve the understanding of the biological character of cutaneous SCC for practicing physicians.
